# Efficacy of Different Types of Exercise on Cognitive Function in People With MCI or Dementia: A Network Meta-Analysis

**DOI:** 10.1093/geroni/igaa057.938

**Published:** 2020-12-16

**Authors:** Qiaoqin Wan, Xiuxiu Huang, Xiaoyan Zhao, Bei Li, Ying Cai, Shifang Zhang

**Affiliations:** 1 Peking University, Beijing, China; 2 Peking university, Beijing, China

## Abstract

With the accelerating progress of population aging, cognitive dysfunction is becoming increasingly prevalent. Exercise, as a promising non-pharmaceutical therapy, showed favorable effects on cognitive function. But which type is the most effective exercise treatment is still unclear. This study compared the efficacy of different types of exercise interventions based on network meta-analysis and aimed to explore the optimal exercise treatment for cognitive decline. The electronic databases of PubMed, Web of Science, Embase, Cochrane Central Register of Controlled Trials, SPORTDiscus, PsycInfoy, and OpenGrey were searched from inception to September 2019. We only included randomized controlled trials that examined the effectiveness of exercise interventions in people with MCI or dementia. Primary outcomes were global cognition, executive function and memory function. Standard mean difference (SMD) and its 95% confidence interval (CI) were calculated to estimate the effect sizes. Finally, 73 articles with 5748 participants were included. The results showed all kinds of exercise interventions were effective on global cognition and resistance exercise was probably the most effective exercise treatment to prevent the decrease of global cognition (SMD=1.05, 95%CI 0.56-1.54), executive function (SMD=0.85, 95%CI 0.21-1.49) and memory function (SMD=0.32, 95%CI 0.01-0.63) for people with cognitive dysfunction. Subgroup analysis revealed multi-component exercise showed more favorable effects on global cognition (SMD=0.99, 95%CI 0.44-1.54) and executive function (SMD=0.72, 95%CI 0.06-1.38) in people with MCI. In conclusion, resistance exercise tended to be the optimal exercise type for people with cognitive dysfunction, especially for people with dementia. And multi-component exercise also should be recommended for people with MCI.

